# The Impact of Disease-Modifying Antirheumatic Drugs on In-Hospital Outcomes of Patients With COVID-19: A Retrospective Cohort Study and Literature Review

**DOI:** 10.7759/cureus.102637

**Published:** 2026-01-30

**Authors:** Vasiliki Tasouli-Drakou, Daniela Rodriguez, Viktoria Krutikova, Abbas Mohammadi, Hossein Akhondi

**Affiliations:** 1 Internal Medicine, University of Nevada, Las Vegas, School of Medicine, Las Vegas, USA; 2 Internal Medicine, Valley Health System, Las Vegas, USA; 3 Infectious Diseases, Harvard Medical School, Boston, USA; 4 Internal Medicine, University of Nevada, Reno School of Medicine, Reno, USA

**Keywords:** covid-19, disease-modifying antirheumatic drugs, dmard, immunomodulation, inflammatory diseases, retrospective study

## Abstract

Objective

Disease-modifying antirheumatic drugs (DMARDs) are widely used to treat autoimmune diseases. However, little is known regarding whether they influence in-hospital outcomes in patients admitted with COVID-19.

Methods

A retrospective cohort study was conducted in two Las Vegas, Nevada, tertiary referral hospitals to evaluate whether the use of DMARD in patients with COVID-19 influences in-hospital outcomes. Data were retrieved from electronic health records of adult patients with COVID-19 who were admitted over six months. Patients were divided into two groups: those who were actively receiving DMARD therapy when they developed COVID-19 (n = 14) and those who were not (n = 553). Primary (mortality rates) and secondary endpoints (type of visit (emergency department versus inpatient admission), need for mechanical ventilation, ICU upgrades, and length of hospital stay) were compared among the two groups.

Results

The two groups had similar baseline characteristics such as age, sex, race, body mass index, and ethnicity. Although differences existed between the groups’ outcomes, these differences were not statistically significant. Mortality rates (p = 0.36), emergency room visit rates ( p = 0.14), ICU admission rate (p = 0.8), total length of hospital stay (8 days vs. 2.8 days, p = 0.2), and ventilator use (p = 0.5) were not statistically significant when comparing the DMARD and non-DMARD groups.

Conclusion

Our study indicates that both non-DMARD and DMARD groups had similar outcomes after contracting COVID-19 without any statistically significant differences. Although not statistically significant, the observation of reduced mortality, admissions, and ventilator use in the DMARD group compared to the non-DMARD group is hypothesis-generating and should be explored in future studies.

## Introduction

The coronavirus disease 2019 (COVID-19) pandemic presented unprecedented challenges to the medical field, characterized by significant uncertainties and a rapidly evolving understanding of the disease [1,2]. From its initial outbreak, healthcare systems worldwide faced immense pressure as they worked to manage an influx of critically ill patients, with scientists and clinicians racing to understand the virus's pathophysiology, transmission, and optimal treatment strategies. Amidst this crisis, attention was turned to the potential impact of pre-existing medical conditions and treatments on the course and outcomes of COVID-19 infections, including immunosuppressive therapies. One such group of treatments, i.e., disease-modifying antirheumatic drugs (DMARDs), is widely used to treat autoimmune diseases such as rheumatoid arthritis, lupus, and psoriatic arthritis [3,4]. These medications were designed to modulate the immune response, often by dampening overactive immune cells and inflammatory processes [3].

Given the critical role of the immune system in the body's response to infections, there has been growing interest regarding how immunosuppressive treatments, particularly DMARDs, might influence the severity of viral infections like COVID-19, which are associated with significant inflammation [5]. The immunomodulatory effect of DMARDs could theoretically alter the course of the disease, affecting outcomes such as disease severity, need for intensive care, or even mortality. The use of DMARDs in COVID-19 patients has sparked a broad range of inquiries into their potential impact on patient outcomes [6]. For example, studies have shown that cluster of differentiation 20 (CD20) and interleukin (IL)-12/23 inhibitors can increase the risk of post-acute sequelae of COVID-19 in systemic autoimmune rheumatic disease (SARD) patients [3]. Moreover, a study by Dernoncourt et al. using World Health Organization (WHO) data showed that tumor necrosis factor (TNF) inhibitors raised COVID-19 risk in inflammatory rheumatic disease (IRD) patients [7]. In contrast, tocilizumab (an anti-IL-6 agent) and Janus kinase (JAK) inhibitors decreased the risk. A study of 174 SARD COVID-19 survivors reported that 51% of them had DMARD disruptions, while 45% had prolonged symptoms [4]. These findings highlight the varying impacts of different DMARDs on COVID-19 outcomes in SARD and IRD patients, emphasizing the need for careful management of immunosuppressive therapies during treatment.

Our study aims to contribute to the growing body of knowledge by investigating whether DMARD use influences key clinical outcomes in hospitalized patients with COVID-19, specifically in the southern Nevada population. Furthermore, it seeks to determine whether DMARD use impacts mortality rates, emergency department (ED) versus floor admission rates, the need for mechanical ventilation, intensive care unit (ICU) upgrades, and the length of hospital stay. By examining these outcomes, the study hopes to provide valuable insights into the possible role of immunosuppressive therapies in managing COVID-19.

## Materials and methods

This retrospective cohort study was conducted at two tertiary care hospitals in Las Vegas, Nevada. Data were collected from electronic health records of patients admitted between January 1, 2024, and June 30, 2024. After review by the Institutional Review Board (IRB) Committee of Touro University Nevada (studies conducted at the hospitals in which our retrospective study took place go through the IRB committee of Touro University Nevada, an academic partner), as this was a retrospective non-experimental study, an IRB exemption determination was provided.

Patients aged 18 years or greater with confirmed severe acute respiratory syndrome coronavirus 2 (SARS-CoV-2) infection via reverse-transcriptase polymerase chain reaction (RT-PCR) were included. A total of 569 patient identification numbers were generated, but two were excluded due to missing data. Patients were categorized into two groups: those actively receiving DMARD therapy within the past six months leading to their admission (n = 14) and those not on DMARD (n = 553). Primary outcome endpoints included in-hospital mortality, while secondary endpoints included the proportion of patients with emergency department visits vs. those admitted directly to the floor, ventilator use rates, ICU upgrade rates, and length of hospital stay. Comparisons between groups were performed using chi-square tests for categorical variables and t-tests for continuous variables. Statistical significance was at p < 0.05.

## Results

Patient characteristics

Common chief complaints reported on admission included cough (n = 198, 35%), dyspnea (n = 96, 17%), fever (n = 85, 15%), sore throat (n = 59, 10%), generalized weakness (n = 46, 8%), altered mental status (n = 19, 3%), syncope (n = 14, 2%), and dizziness (n = 8, 1%). A total of 44 patients (8%) had a history of asthma and chronic obstructive pulmonary disease (COPD).

The mean age of patients on DMARD was 58.64 years (SD = 20.56), compared to 57.32 years (SD = 21.66) for those who were not. Gender distribution differed between groups, with 21% of patients being male in the DMARD group and 38% being male in the non-DMARD group (p = 0.197). Table 1 summarizes the characteristics of the two groups. No statistical differences were noted when comparing the age, body mass index, ethnicity, race, and history of concurrent respiratory problems among these two groups. Eight patients in the non-DMARD group had a reported history of a pertinent autoimmune disease (two patients had a reported history of psoriasis, two had a history of rheumatoid arthritis, and four had a diagnosis of systemic lupus erythematosus), but were not on a DMARD. Name and dosage of the DMARD taken by the DMARD group are listed in Table 2. Six out of the eight DMARD patients who had an inpatient status did not have their DMARD continued during their hospitalization (Table 3).

**Table 1:** Patient characteristics among the two groups. The patient count is listed in parentheses. DMARD: disease-modifying antirheumatic drug; BMI: body mass index; SD: standard deviation; COPD: chronic obstructive pulmonary disease.

	DMARD group	Non-DMARD group	p-value
Age (average in years)	58.64 (SD=20.54)	57.32 (SD=21.66)	0.870
Average BMI (kg/m^2^)	26.70 (SD=5.97)	29.30 (SD=7.66)	0.470
Sex			0.197
Female	78.6% (11)	61.7% (341)	
Male	21.4% (3)	38.3% (212)	
Ethnicity			0.494
Hispanic	21.4% (3)	14.8% (82)	
Non-Hispanic	78.6% (11)	85.2% (471)	
Race			
White	50% (7)	44.5% (246)	0.682
Black or African American	28.6% (4)	24.8% (137)	0.745
Native Hawaiian or Pacific Islander	0	1% (6)	0.695
Asian	0	8.9% (49)	0.243
Unknown or other	21.4% (3)	20.6% (114)	0.778
Alaskan Indian	0	0.2% (1)	0.873
Respiratory comorbidities			
Asthma	0% (0)	3.6% (20)	0.469
COPD	7.1% (1)	4.2% (23)	0.584

**Table 2:** Reported autoimmune conditions, DMARD names, and dosages for the DMARD group. Concurrent steroid use prior to hospitalization was also included. When looking at the reported autoimmune conditions and documented DMARDs, it is very likely that not all autoimmune conditions were documented in the patients’ medical charts. DMARD: disease-modifying antirheumatic drug; NH: non-Hispanic; HI: Hispanic; RA: rheumatoid arthritis; SLE: systemic lupus erythematosus; MTX: methotrexate.

Age (years)	Sex	Reported autoimmune condition	Documented DMARD	Dose of DMARD	Concurrent steroid use prior to hospitalization
71	Female (NH)	RA	Upadacitinib; leflunomide	15 mg/day; 20 mg/day	No
29	Female (NH)	SLE	Hydroxychloroquine	200 mg/day	Yes (prednisone)
91	Female (NH)	RA	MTX; hydroxychloroquine	12.5 mg/weekly; 200 mg/day	No
53	Male (HI)	RA	Hydroxychloroquine	400 mg/day	Yes (prednisone)
29	Male (NH)	Crohn’s	MTX	12.5 mg/weekly	Yes (prednisone)
30	Female (HI)	SLE	Hydroxychloroquine; azathioprine	200 mg day; 50 mg/day	No
77	Female (NH)	RA, SLE	Azathioprine	50 mg/day	No
78	Female (NH)	RA	MTX	2.5 mg/weekly	Yes (methylprednisolone)
65	Male (NH)	SLE	MTX	25 mg/weekly	No
74	Female (NH)	RA	MTX	25 mg/weekly	No
73	Female (HI)	SLE	Hydroxychloroquine	400 mg/day	Yes (prednisone)
48	Female (NH)	Multiple sclerosis	Teriflunomide	14 mg/day	No
40	Female (NH)	RA	MTX	0.7 ml/weekly	No
63	Female (NH)	Possible antisynthetase syndrome	MTX	15 mg/weekly	Yes (prednisone)

**Table 3:** Reported non-autoimmune comorbidities of the DMARD group and continuation of DMARD during hospitalization. DMARD: disease-modifying antirheumatic drug; NH: non-Hispanic; HI: Hispanic; COPD: chronic obstructive pulmonary disease.

Age (years)	Sex	Non-autoimmune comorbidities	Continuation of DMARD during hospitalization
71	Female (NH)	Hypertension, hyperlipidemia, paroxysmal atrial fibrillation	Yes
29	Female (NH)	Hypertension, seizures, deep vein thrombosis	Yes
91	Female (NH)	Lymphoma, deep vein thrombosis	Yes
53	Male (HI)	Follicular lymphoma, hyperlipidemia	No
29	Male (NH)	Colorectal cancer, stroke	Yes
30	Female (HI)	Fibromyalgia	No – Emergency visit
77	Female (NH)	None	No – Emergency visit
78	Female (NH)	Hyperlipidemia, hypertension	No – Emergency visit
65	Male (NH)	Coronary artery disease, hyperlipidemia, hypertension, sleep apnea	No – Emergency visit
74	Female (NH)	Hypertension, hyperlipidemia, COPD	Yes
73	Female (HI)	Type 2 diabetes mellitus, hyperlipidemia, hypertension	No – Emergency visit
48	Female (NH)	None	No – Emergency visit
40	Female (NH)	None	No
63	Female (NH)	Type 2 diabetes mellitus, sleep apnea	Yes

In this retrospective study, only one out of the 14 DMARD patients was on a non-conventional DMARD; this individual with rheumatoid arthritis presented with the concurrent use of upadacitinib, a targeted synthetic DMARD and JAK inhibitor, and leflunomide. This 71-year-old patient spent 10 days at the hospital and did not require an upgrade to the ICU. As a result, no t-test p-value with respect to the length of ICU and total hospital stay could be calculated when comparing this individual with other rheumatoid arthritis patients on conventional DMARD therapy. Thus, no extrapolation could be made to suggest that rheumatoid arthritis patients receiving biologic DMARD appear to have worse COVID-19 outcomes than those prescribed conventional DMARDs, as suggested by some recent data [8].

Because a prior study by Sechrist et al. had suggested that concurrent use of corticosteroids and DMARD was associated with a significantly increased risk of COVID-19 hospitalizations, concurrent use of steroids such as prednisone prior to the hospitalization was recorded for DMARD patients, as seen in Table 2 [9]. To better evaluate whether DMARD patients with concurrent steroid use had worse clinical outcomes compared to DMARD-only individuals, p-values were calculated and presented in Table 4. No statistically significant differences were found with respect to length of hospital or ICU stay between the two DMARD groups. However, statistically significant differences were seen when looking at the race of individuals, with White patients being less likely to be prescribed steroids.

**Table 4:** Comparison of clinical outcomes between DMARD individuals with concomitant use of steroids and DMARD and those without. DMARD: disease-modifying antirheumatic drug; SD: standard deviation; *: statistically significant p-value.

	Concurrent steroid and DMARD use	DMARD use only	p-value
Average length of hospital stay (days)	11 (SD=20.43)	0.33 (SD=0.82)	0.724
Average length of ICU stay (days)	5.75 (SD=8.73)	0	0.264
Ethnicity			0.375
Hispanic	33.3% (2)	12.5% (1)	
Non-Hispanic	66.7% (4)	87.5% (7)	
Race			
White	16.7% (1)	75% (6)	0.003*
Black or African American	50% (3)	12.5% (1)	0.105
Unknown or other	33.3% (2)	12.5% (1)	0.347

Primary outcome

No in-hospital mortality was reported among patients in the DMARD group, while 14 patients passed away in the non-DMARD group. This was not statistically significant (p = 0.36).

Secondary outcomes

ED-only visits (meaning patients not getting admitted to the floor) were fewer in the DMARD group (40%) compared to the non-DMARD group (58.5%), but this difference was not statistically significant (p = 0.14). Ventilator use did not differ significantly (p = 0.5) between the groups. No patient was placed on a ventilator in the DMARD group, while 13 non-DMARD patients were placed on a ventilator. The average number of days on a ventilator was 5.77.

**Figure 1:**
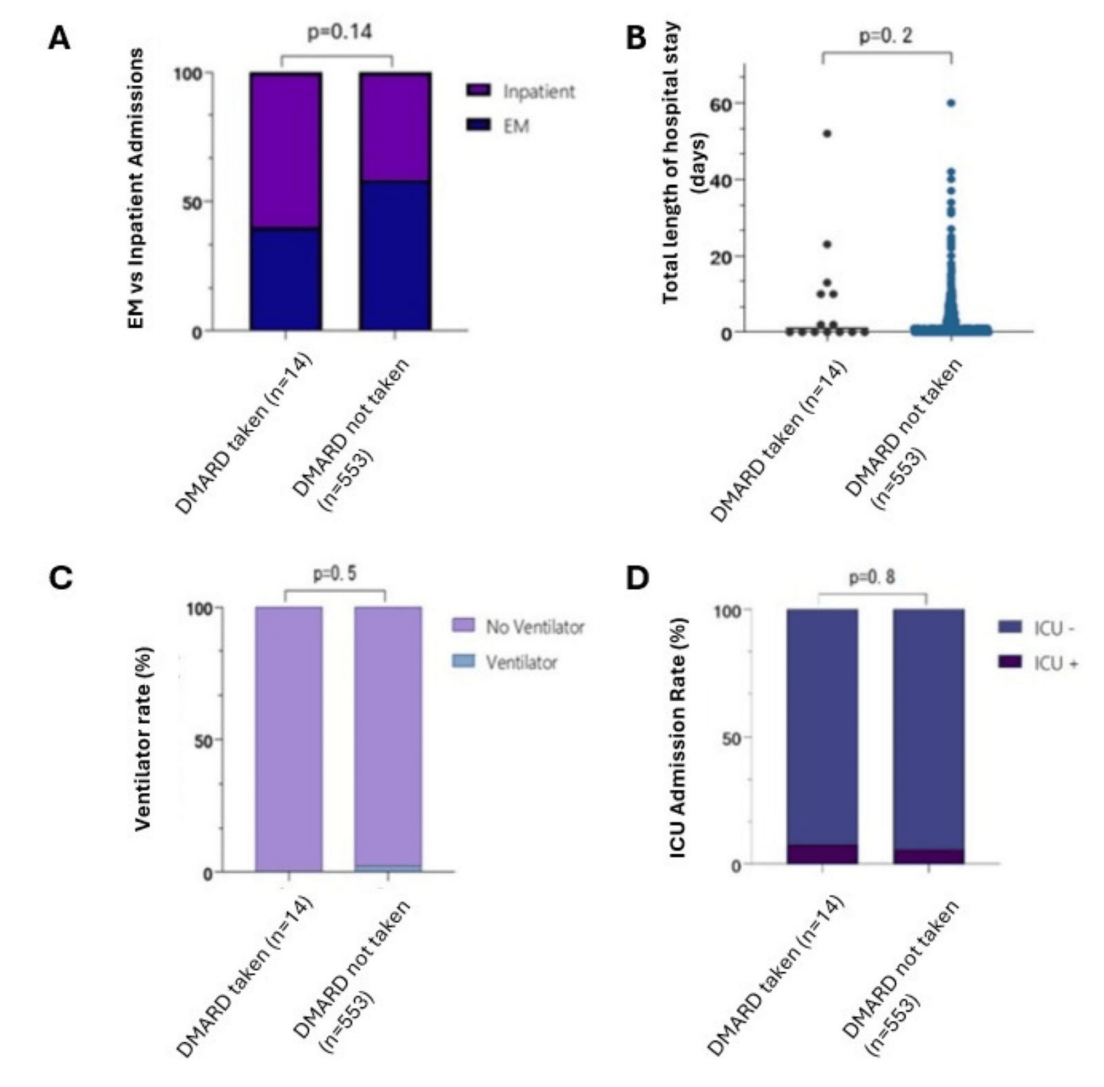
Secondary outcome endpoints among the DMARD and non-DMARD groups. No statistically significant differences were observed when comparing the type of admission (A; emergency versus inpatient), total length of hospital stay (B), and rates of ventilator use (C) and ICU admission (D). DMARD: disease-modifying antirheumatic drug; EM: emergency medicine; ICU: intensive care unit.

Among the DMARD group, only one patient was upgraded to the ICU for a total duration of two days, while 31 patients were upgraded to the ICU in the non-DMARD group for an average duration of 6.52 days. Again, no statistical significance was observed between the two groups (p = 0.8). Finally, the length of hospital stay was not statistically significant among the two groups (average of eight days for the DMARD group and 2.84 days for the non-DMARD group, p = 0.2). Secondary endpoint outcomes are summarized in Figure 1. Furthermore, although not a secondary endpoint, no statistical difference between the DMARD and non-DMARD groups was observed regarding the use of additional oxygen supplementation in the form of nasal cannula, high-flow nasal cannula, and bilevel positive airway pressure (BIPAP) during their hospitalization (p = 0.70).

## Discussion

In this retrospective cohort study, we investigated the impact of DMARD use on outcomes in patients hospitalized with COVID-19, including mortality, ED versus direct floor admission rates, ventilator use, and length of stay. Our findings indicate no statistically significant differences between patients on DMARD and those not on DMARD for these outcomes.

While mortality was numerically lower in the DMARD group compared to the non-DMARD group, the difference did not reach statistical significance, possibly due to the small sample size of the DMARD cohort. Similarly, the rate for standalone emergency department visits in the DMARD group and the comparable ventilator use, ICU admissions, and length of stay between the groups possibly allude to the fact that DMARDs do not exacerbate disease severity [10]. While due to the small sample size of DMARD patients and the exploratory nature of the study, suggestive trends may not be extrapolated, the observation of reduced mortality, admissions, and ventilator use in the DMARD group compared to the non-DMARD group is hypothesis-generating and should be explored in future studies. Our study also found that among DMARD users, White patients were less likely to be concurrently prescribed steroids compared to non-White patients, something that parallels studies that have supported this finding [11]. It is worth also noting that long-term glucocorticoid use has been associated with worse health outcomes among patients with systemic lupus erythematosus, a condition that is more prevalent in African Americans [12]. Therefore, future studies and clinical trials should evaluate patterns of glucocorticoid use in an effort to help address and mitigate disparities in steroid-related adverse outcomes.

The above findings nonetheless parallel emerging evidence suggesting that some immunosuppressive therapies may have neutral or helpful effects in COVID-19 due to their role in modulating hyperinflammatory responses. A study by Ward et al. that investigated the effect of immunosuppressants on the prognosis of SARS-CoV-2 infection found that although the use of glucocorticoids was associated with increased risks of hospital admission and death, exposure to TNF, interleukin, or calcineurin inhibitors, as well as hydroxychloroquine or chloroquine, was not associated with an increased risk of hospitalization, ICU admission, or death [13]. This finding is notably reaffirmed when considering the meta-analysis by Tassone et al., which showed that immunosuppressed patients are not at an increased risk of COVID-19 infection [10].

Studies showing potential benefits in DMARD patients on TNF and JAK inhibitors

A study by Salesi et al. found that patients with rheumatoid arthritis (RA) and seronegative spondyloarthropathies (SpA) treated with TNF-α blockers (infliximab, adalimumab, and etanercept) had a significantly decreased risk of developing COVID-19 [14]. Moreover, a study by Gianfrancesco et al. that evaluated individuals with RA found that RA patients receiving anti-TNF medications and who tested positive for COVID-19 had reduced odds of hospitalization [15].

JAKs are tyrosine kinases that, upon binding cytokines to their specific receptors, phosphorylate both the receptors and the recruited STAT proteins and, through a series of reactions, help regulate gene transcription of cytokines [16]. These cytokines, including IL-6, IL-12, and IL-23, are considered to play a role in inflammation, B- and T-cell proliferation, and differentiation. A meta-analysis by Patoulias et al. found similar results to the study by Dernoncourt et al. [7], with patients utilizing JAK inhibitors (baricitinib, ruxolitinib, tofacitinib, and nezulcitinib) having significantly reduced risks for COVID-19-associated deaths and risk for mechanical ventilation or extracorporeal membrane oxygenation (ECMO) initiation [17].

Studies showing equality of endpoints for DMARD patients on IL-17 and IL-23 inhibitors

COVID-19 infections are characterized by inflammation and the release of cytokines, the latter of which, if severe, can lead to cytokine-induced lung injuries [18]. It is known that cytokines of the IL-17 family can prompt the apoptosis of alveolar epithelial cells and the progression to pulmonary fibrosis [19]. They can be stimulated by a variety of other pro-inflammatory cytokines, including transforming growth factor beta (TGF-β), IL-23, and IL-6. For this reason, it has been suggested that therapies targeting the IL-17 cytokine family could be a promising treatment in patients with COVID-19. A meta-analysis by Liu et al. that evaluated psoriasis patients treated with IL-17 inhibitors found that these patients were not at an increased risk for SARS-CoV-2 infection, COVID-19 hospitalization, and COVID-19 mortality compared to individuals not receiving biologics [20].

This finding was confirmed by the study of Kridin et al., which did not find a significant difference in the rates of COVID-19 incidence, COVID-19-associated hospitalizations, and mortality when comparing psoriasis patients receiving the IL-17 inhibitors secukinumab and ixekizumab with patients who were either on methotrexate or who were not on any systemic or immunomodulatory treatment [21].

IL-6, on the other hand, has been demonstrated to have protective effects against viral replication by stimulating scavenger macrophages and T-cell responsivity, as well as pro-inflammatory effects on immune and endothelial cells in severe disease [22]. Trials comparing the use of tocilizumab, an IL-6 inhibitor, with placebo in hospitalized patients with severe pneumonia due to COVID-19 found that tocilizumab did not result in a significantly better clinical status (i.e., need for ventilation and adverse bleeding, hepatic, and cardiac events) or lower mortality [23,24].

Mixed-results studies on DMARD patients on IL-17 and CD20 inhibitors

Cytokines IL-12 and IL-23, secreted by dendritic cells (DCs) and macrophages in response to recognition of exogenous microbial components, play an important role in promoting T-cell activation and amplification of inflammation [25]. Hence, inhibiting these cytokines could theoretically lead to milder manifestations of COVID-19. A study by Yan et al. found that IL-12/23 or IL-23 inhibitor therapy in psoriatic patients was associated with an increased risk of contracting COVID-19 but not with an increased risk of developing severe disease [26]. The authors reported that using methotrexate, apremilast (a phosphodiesterase-4 inhibitor), TNF-α inhibitors, and interleukin-17 inhibitors, however, did not affect COVID-19 outcomes. Another study by Hu et al., however, indicated that individuals treated with IL-23 inhibitors were more likely to be asymptomatic (i.e., free from fatigue, dyspnea, abnormal sense of smell and taste, and persistent cough) after recovery from COVID-19 compared to patients treated with methotrexate and TNF-α inhibitors [27].

Dernoncourt et al., utilizing data from the WHO database, found that COVID-19 was significantly more frequent among patients with IRDs treated with TNF inhibitors (reporting odds ratio (ROR): 8.31) [7]. In contrast, tocilizumab (anti-IL-6) and JAK inhibitors showed a lower COVID-19 risk in rheumatoid arthritis patients (RORs: 0.12 and 0.33, respectively). The findings suggest that TNF inhibitors may increase COVID-19 susceptibility in IRD patients, while anti-IL-6 and JAK inhibitors may have a safer profile, warranting further investigation into their use during COVID-19 infection. This finding may come to some surprise, as TNF-α, acting by binding to two receptors (TNFR1 and TNFR2), which are expressed by all human tissues and by immune cells, respectively, influences biological functions such as inflammation, tissue degeneration, host defense, cell proliferation, and cell survival [28]. Nonetheless, TNF-α-mediated inflammation may also lead to detrimental tissue damage and lung fibrosis [29]. A cause for the discrepancy between these studies may be the differences in need for immunosuppression, not only between IRD and non-IRD patients but also among different IRD subgroups. For example, patients with inflammatory bowel disease have a more significant need for immunosuppression compared to patients with rheumatoid arthritis [7].

Additionally, although it is generally believed that patients who have received anti-CD20 therapies may be at an increased risk of having prolonged viral infections and more severe clinical outcomes due to B-cell depletion, a 2023 study by Kasten et al. found that in patients on anti-CD20 therapies who were hospitalized for COVID-19 and who received treatment (i.e., remdesivir, glucocorticoids, and convalescent plasma), the duration from receipt of last dose of anti-CD20 therapy did not correlate with outcomes [30]. The study did confirm that these patients, however, exhibited a high rate of rehospitalization for COVID-19.

Studies showing worsening outcomes in DMARD patients on IL-12/23 and CD20 inhibitors

Venkat et al. found that among 510 patients with SARD and COVID-19, CD20 inhibitor users had significantly higher odds of developing post-acute sequelae of COVID-19 (PASC), defined as COVID-19 symptoms persisting for 28 days after infection, and had fewer symptom-free days compared to conventional synthetic DMARD users [3]. However, no statistically significant differences in symptom-free days were noted after 90 days. Moreover, the authors found that IL-12/23, IL-17A, and IL-23 inhibitor users also showed increased PASC risk. These findings suggest that specific DMARD classes, particularly CD20 and IL-12/23 pathway inhibitors, may elevate PASC risk in SARD patients.

Our results and study limitations

Most patients in our study were on methotrexate and hydroxychloroquine, with a few taking azathioprine, teriflunomide, leflunomide, and upadacitinib (a JAK inhibitor). When looking at their respective mechanisms of action, teriflunomide is an inhibitor of dihydroorotate-dehydrogenase (DHODH), an enzyme involved in the de novo synthesis of pyrimidines in rapidly proliferating T-lymphocytes and B-lymphocytes [31]. Teriflunomide, like its parent drug leflunomide (converted into teriflunomide in the intestinal mucosa), can diminish inflammatory responses by reducing the number of available T-cells [32]. Azathioprine, a purine analog, can become incorporated into replicating DNA and block purine synthesis, a process necessary for lymphocyte proliferation. Moreover, hydroxychloroquine can interfere with lysosomal activity and autophagy and disrupt signaling pathways involved in cytokine production [33].

A case series of 18 teriflunomide-treated multiple sclerosis patients diagnosed with COVID-19 showed that none of the patients required hospitalization and that all had recovered within seven to 14 days, demonstrating that the use of teriflunomide therapy during COVID-19 infections is safe and that it should not be discontinued [34]. These findings are further supported by a case series by Scharrer et al. that included patients with inflammatory bowel disease receiving azathioprine [35].

Regarding hydroxychloroquine, a study by Putman et al. found that using hydroxychloroquine in patients hospitalized with COVID-19 was not associated with a mortality benefit [36]. This study was done in patients who were given hydroxychloroquine as a treatment for COVID-19 infection due to its ability to inhibit SARS-CoV-2 by altering the glycosylation of the angiotensin-converting enzyme 2 (ACE2) receptor. A review article by Konig et al. drew attention to the fact that baseline use of hydroxychloroquine in systemic lupus erythematosus was not found to preclude SARS-CoV-2 infection and severe COVID-19 clinical outcomes [37].

When it comes to methotrexate, it has been shown that patients with immune-mediated inflammatory diseases on methotrexate receiving COVID-19 vaccinations have lowered responses of T-cells and antibodies compared to controls [38]. Nevertheless, a study by Ganjei et al. comparing COVID-19 severity between patients on methotrexate at baseline and patients with normal immune function, showed that patients taking methotrexate experienced a milder disease course (such as fever, cough, and dyspnea), with the authors alluding to the presence of lowered TNF-α and IL-6 levels and increased T regulatory cells as a possible justification for the finding of these results [39].

When it comes to our study, it is certainly not without limitations. It is limited by the small size of the DMARD group (n = 14), which restricts the statistical power to detect meaningful differences. Factors that may have influenced this finding include limited access to rheumatology specialists in Las Vegas due to a patient's socioeconomic status or specialist shortage, and medications not appearing under dispense reports due to patients visiting from out of state, failing to pick up a prescription, having out-of-network pharmacies, or having pended prior authorizations or insurance denials. Other reasons include failing to mark that patients are taking DMARD therapies under home medications at the time of their hospital visit (especially ED admissions), injections or infusions given at a hospital or in-office, patient preferences, and finally, provider discontinuation of a DMARD medication. Given these possible factors and the fact that this is a retrospective study relying on documented data, it is highly probable that the number of patients on DMARD was under-reported.

The retrospective design and reliance on data from two tertiary care centers further limit the generalization of our findings. Non-documented confounding variables such as patients’ insurance status and access to specialist care, behavioral and lifestyle factors (i.e., smoking and medication adherence), as well as hospital type (tertiary care center vs. academic institution) might further influence the observed findings. Our study also chose to focus on respiratory comorbidities (COPD and asthma) for both groups, given that most patients presented with upper respiratory symptoms. Indeed, the presence of COPD in patients diagnosed with COVID-19 has been associated with increased odds of hospitalization, ICU admission, and mortality in the literature [40]. This finding, however, is not supported in the meta-analysis by Sunjaya et al., which compared COVID-19 patients with and without a history of COPD [41]. Nonetheless, non-autoimmune comorbidities were recorded for the DMARD group, as seen in Table 3. Although no statistically significant differences in clinical outcomes were observed between the DMARD and non-DMARD groups, had other non-respiratory comorbidities for the non-DMARD group also been recorded, it is possible that no statistically significant differences may have been observed.

Moreover, the COVID-19 vaccination status of individuals was not recorded, which could have been associated with significantly lower hospitalization and mortality risks [42]. Similarly, disease activity (i.e., assessing the erythrocyte sedimentation rate and C-reactive protein levels) among DMARD patients was also not recorded, something that could have provided more insight into the inflammatory burden of individuals and their hospitalization course progression. At the same time, drug dosage heterogeneity was seen in our DMARD group, especially among patients with the same autoimmune conditions, such as RA and lupus. While understandably different individuals carry varying degrees of inflammatory burden, this increased within-group variability could have potentially influenced exposure responses and safety conclusions. The continuation of DMARD for six of the eight DMARD patients during their inpatient hospitalization may have also influenced the course of treatment. These individuals were nonetheless not compared with each other. Future studies with more extensive multicenter cohorts are needed to confirm these results and explore potential mechanisms underlying the interaction between DMARD use and COVID-19 outcomes.

## Conclusions

To conclude, both hospitalized DMARD and non-DMARD users who contracted COVID-19 presented with similar clinical outcomes (i.e., total length of hospital and ICU stay and ventilator use). The statistically significant p-value regarding White individuals on DMARD therapy being less likely to receive concurrent use of steroids is hypothesis-generating and should be explored in future studies.
